# Contribution of proton leak to oxygen consumption in skeletal muscle during intense exercise is very low despite large contribution at rest

**DOI:** 10.1371/journal.pone.0185991

**Published:** 2017-10-18

**Authors:** Bernard Korzeniewski

**Affiliations:** Faculty of Biochemistry, Biophysics and Biotechnology, Jagiellonian University, Kraków, Poland; University of Birmingham, UNITED KINGDOM

## Abstract

A computer model was used to simulate the dependence of protonmotive force (Δp), proton leak and phenomenological (involving proton leak) ATP/O_2_ ratio on work intensity in skeletal muscle. Δp, NADH and proton leak decreased with work intensity. The contribution of proton leak to oxygen consumption ($\dot{V}O_{2}$) decreased from about 60% at rest to about 3 and 1% at moderate and heavy/severe exercise, respectively, while the ATP/O_2_ ratio increased from 2.1 to 5.5 and 5.7. A two-fold increase in proton leak activity or its decrease to zero decreased/increased the ATP/O_2_ ratio by only about 3 and 1% during moderate and heavy/severe exercise, respectively. The low contribution of proton leak to $\dot{V}O_{2}$ in intensively working skeletal muscle was mostly caused by a huge increase in ATP usage intensity during rest-to-work transition, while OXPHOS, and thus oxidative ATP supply and $\dot{V}O_{2}$ related to it, was mostly stimulated by high each-step activation (ESA) of OXPHOS complexes. The contribution of proton leak to $\dot{V}O_{2}$ and ATP/O_2_ ratio in isolated mitochondria should not be directly extrapolated to working muscle, as mitochondria lack ESA, at least in the absence of Ca^2+^, and therefore $\dot{V}O_{2}$ cannot be elevated as much as in intact muscle.

## Introduction

Proton leak across the inner mitochondrial membrane [1–4] leads to dissipation of the protonmotive force (Δp) that is not coupled with ATP synthesis or ATP, ADP and P_i_ transport across this membrane. The mechanism of H^+^ ions flow across the membrane is still not fully understood [1–4]. The intensity of the proton leak through the inner mitochondrial membrane (vLK) depends steeply (non-ohmic dependence) on the protonmotive force Δp [5–9]. Proton leak can be constitutive (basal proton conductance) and regulated (inducible proton conductance catalyzed by uncoupling proteins, UCPs) [1–4]. Thyroid hormones elevate the constitutive proton leak [10]. Basal vLK is inversely proportional to body mass [11] and is higher in warm-blooded animals than in cold-blooded animals of the same mass [12]. Proton leak can be induced by reactive oxygen species (ROS) acting through UCPs [2,13].

The contribution of proton leak to $\dot{V}O_{2}$ in perfused resting rat skeletal muscle was estimated by Rolfe and Brand to be about 60% at 37°C [8]. On the other hand, Marcinek and co-workers [14] estimated, on the basis of *in vivo* spectroscopic measurements, that there is essentially no proton leak in resting skeletal muscle.

It has been proposed that the so-called each-step activation (ESA) mechanism is a major mechanism of the regulation of OXPHOS during work transitions [15–21]. According to this mechanism, NADH supply, glycolysis and all OXPHOS complexes (complex I, complex III, complex IV, ATP synthase, ATP/ADP carrier and P_i_ carrier) are directly activated by some cytosolic factor(s)/mechanism(s) in parallel with the activation of ATP usage by Ca^2+^.

The present study is aimed to investigate in the theoretical way the contribution of vLK-related $\dot{V}O_{2}$ to total $\dot{V}O_{2}$ and the phenomenological (involving proton leak) ATP/O_2_ ratio at different work intensities in skeletal muscle. The effect of the proton leak activity on these variable values is also studied. The dependence of total $\dot{V}O_{2}$, ATP synthesis-related $\dot{V}O_{2}$, Δp, NADH and proton leak-related $\dot{V}O_{2}$ on work intensity is simulated. It is hypothesized that the total $\dot{V}O_{2}$ and ATP supply-related $\dot{V}O_{2}$ will rise linearly with ATP demand activity (work intensity), while Δp and, consequently, leak-related $\dot{V}O_{2}$ will drop. As a result, the contribution of vLK-related $\dot{V}O_{2}$ to overall oxygen consumption will decrease very significantly, while the phenomenological ATP/O_2_ ratio will increase. It is also expected that an increase in proton leak activity (rate constant) will increase the contribution of vLK-related $\dot{V}O_{2}$ to total $\dot{V}O_{2}$, decrease the phenomenological ATP/O_2_ ratio and Δp, while reduction of the proton leak activity to zero will have the opposite effect. It is expected that the presence of high each-step activation (ESA) of OXPHOS, essentially increasing its capacity for ATP supply and allowing to match the highly elevated ATP usage activity, leads to a low contribution of vLK-related $\dot{V}O_{2}$ to total $\dot{V}O_{2}$, much lower than at rest, and thus to a high phenomenological ATP/O_2_ ratio (very close to the mechanistic, not involving proton leak, ATP/O_2_ ratio) in intensively working skeletal muscle.

## Theoretical methods

The previously-developed computer model of the cell bioenergetic system in skeletal muscle [22,23] was used for theoretical studies. This model comprises explicitly: oxidative phosphorylation complexes (complex I, complex III, complex IV, ATP synthase, ATP/ADP carrier, P_i_ carrier), NADH supply block, ATP usage, proton leak, creatine kinase (CK) system, adenylate kinase (AK), proton efflux/influx to/from blood, (anaerobic) glycolysis. This model has been widely validated through comparison of computer simulations with experimental results [15–23].

**Fig 1:**
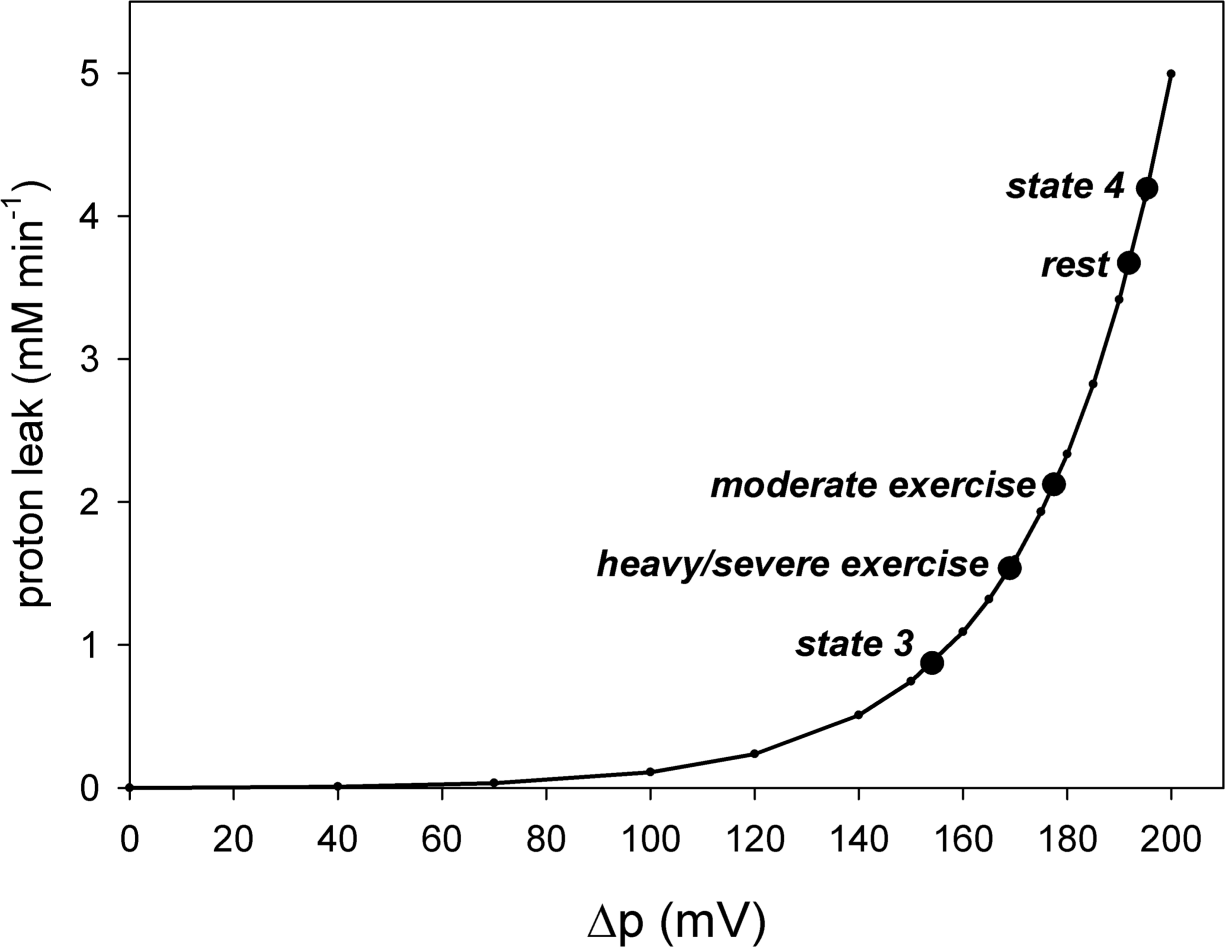
Dependence of proton leak intensity (vLK) on protonmotive force (Δp). State 4, state 3, rest, moderate work and heavy/severe work in skeletal muscle are indicated. vLK in all states is scaled for intact skeletal muscle. Simulations were made using the model version for intact skeletal muscle, as described in the text.

The intensity of proton leak in skeletal muscle is described by the following kinetic equation:

$$v_{LK}=k_{LK1}\cdot \left(e^{k_{LK2}\cdot \Delta p}-1\right)$$ (1)

where k_LK1_ = 2.5 μM min^-1^ and k_LK2_ = 0.038 mV^-1^. The dependence of proton leak intensity on Δp is strongly non-ohmic as presented in Fig 1. This dependence was extracted from different experimental data (see e.g., [5–9]) in order to represent them at least semi-quantitatively (anyway, these data differ to some extent one from another).

In order to produce Fig 1, state 4 and state 3 that are present in isolated mitochondria, but absent in intact skeletal muscle had to be simulated. A state corresponding to state 4 in isolated mitochondria can be induced in intact skeletal muscle by oligomycin (inhibitor of ATP synthase) administration. On the other hand, state 3 and state 3.5 (in the absence of ESA) in intact skeletal muscle are in a sense hypothetical, because they would need turning off of ESA to be reached. At the present state of knowledge we do not know how to do this in intact skeletal muscle, because ESA is present in intact skeletal muscle, but absent in state 3 and state 3.5 in isolated mitochondria (at least in the absence of Ca^2+^ ions). Within the model a state corresponding to state 4 was reached in computer simulations by a decrease in the activity (rate constant) of ATP usage to zero. A state analogous to state 3 in isolated mitochondria was reached by an increase in this constant to saturating (for OXPHOS) values in the absence of the each-step activation (ESA) of OXPHOS complexes. In these simulations, CK and glycolysis were ‘switched off’ [18] (this concerns only the simulations presented in Fig 1). The version of the model for intact skeletal muscle rather than the version for isolated mitochondria [18] was used in order to scale the respiration rate in particular states to intact skeletal muscle respiration. The moderate work state and heavy/severe work state were simulated as described below. Generally, the theoretical points shown in Fig 1 were obtained by simulation of particular states and recording vLK and Δp in them. The line corresponding to the general vLK-Δp dependence represents simply Eq 1.

The simulations carried out and presented in this study concern steady-state variable values and not time courses of variable values. The basal or initial steady-state for all simulations was rest state with ‘standard’ parameter values. In each simulation one or a few parameter values were changed, as described below, and then the system was allowed to approach a new steady-state, in which new variable values were recorded.

**Fig 2:**
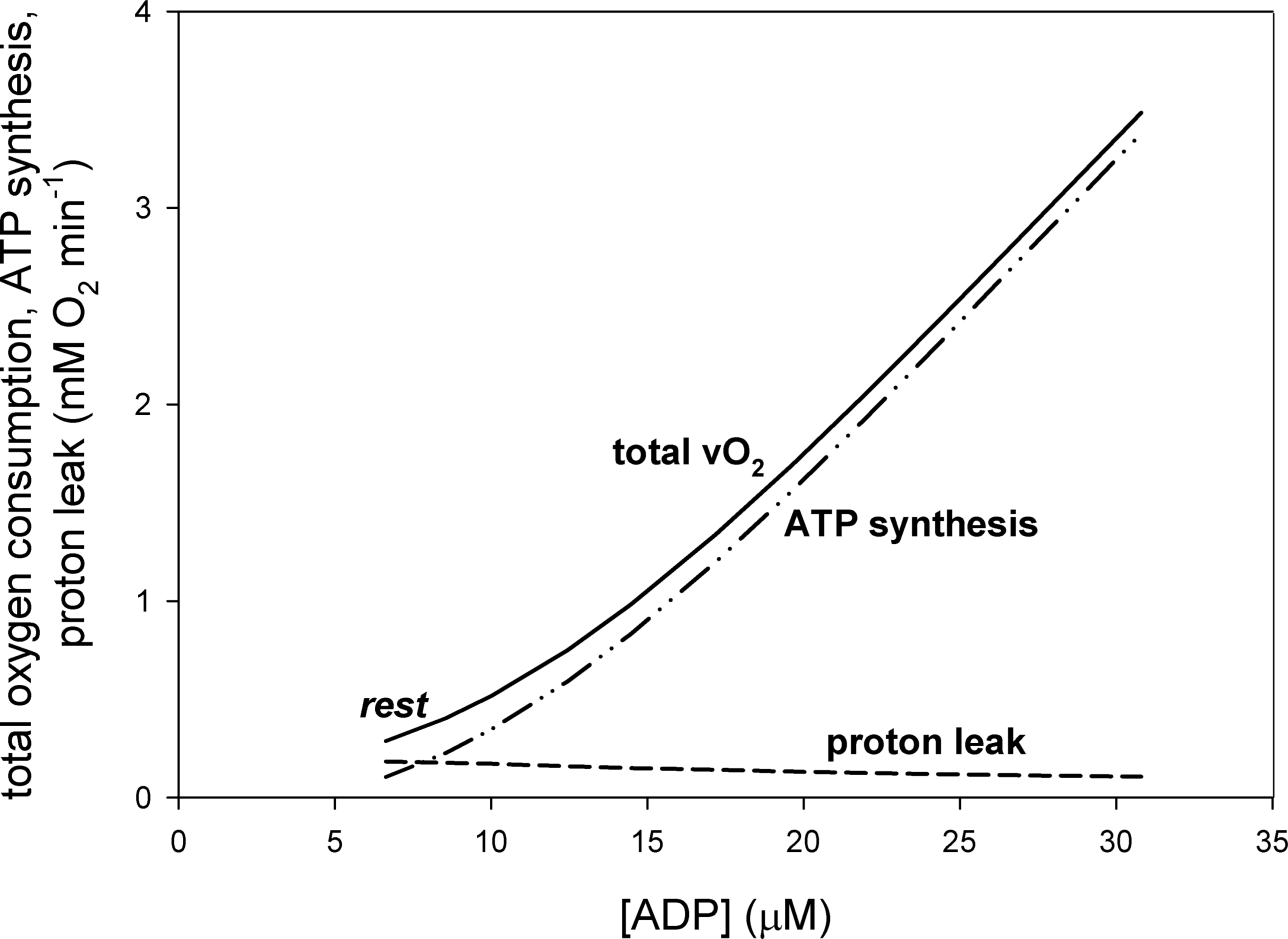
Dependence of total oxygen consumption ($\dot{V}O_{2}$), $\dot{V}O_{2}$ related to proton leak and $\dot{V}O_{2}$ related to ATP synthesis on ADP concentration.

**Fig 3:**
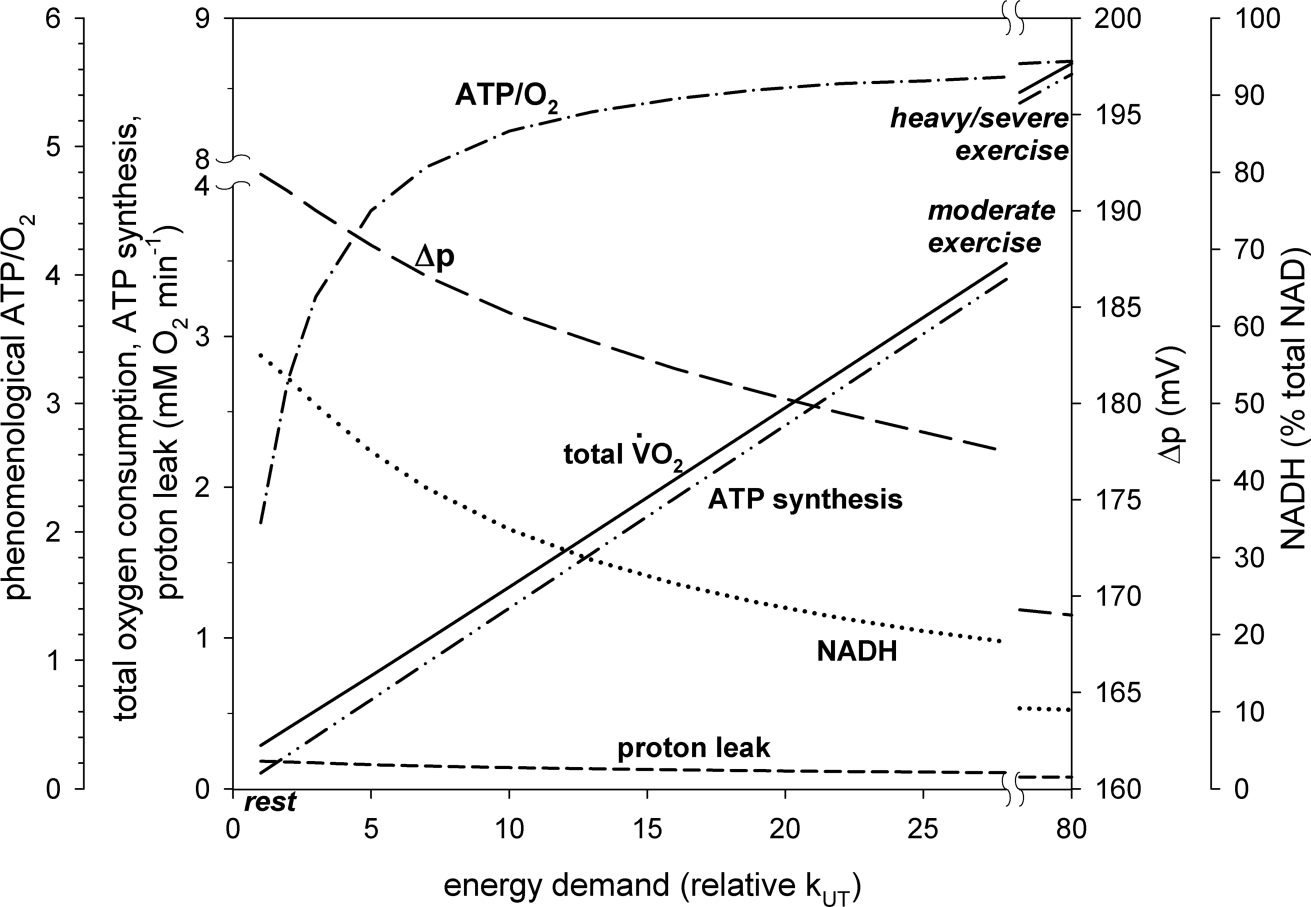
Dependence of total oxygen consumption ($\dot{V}O_{2}$), $\dot{V}O_{2}$ related to proton leak, $\dot{V}O_{2}$ related to ATP synthesis, Δp, NADH (% of total NAD) and phenomenological ATP/O_2_ ratio on relative activity of ATP usage (relative rate constant, k_UT_, or its activation in relation to rest, A_UT_) in skeletal muscle for ‘standard’ proton leak activity.

**Fig 4:**
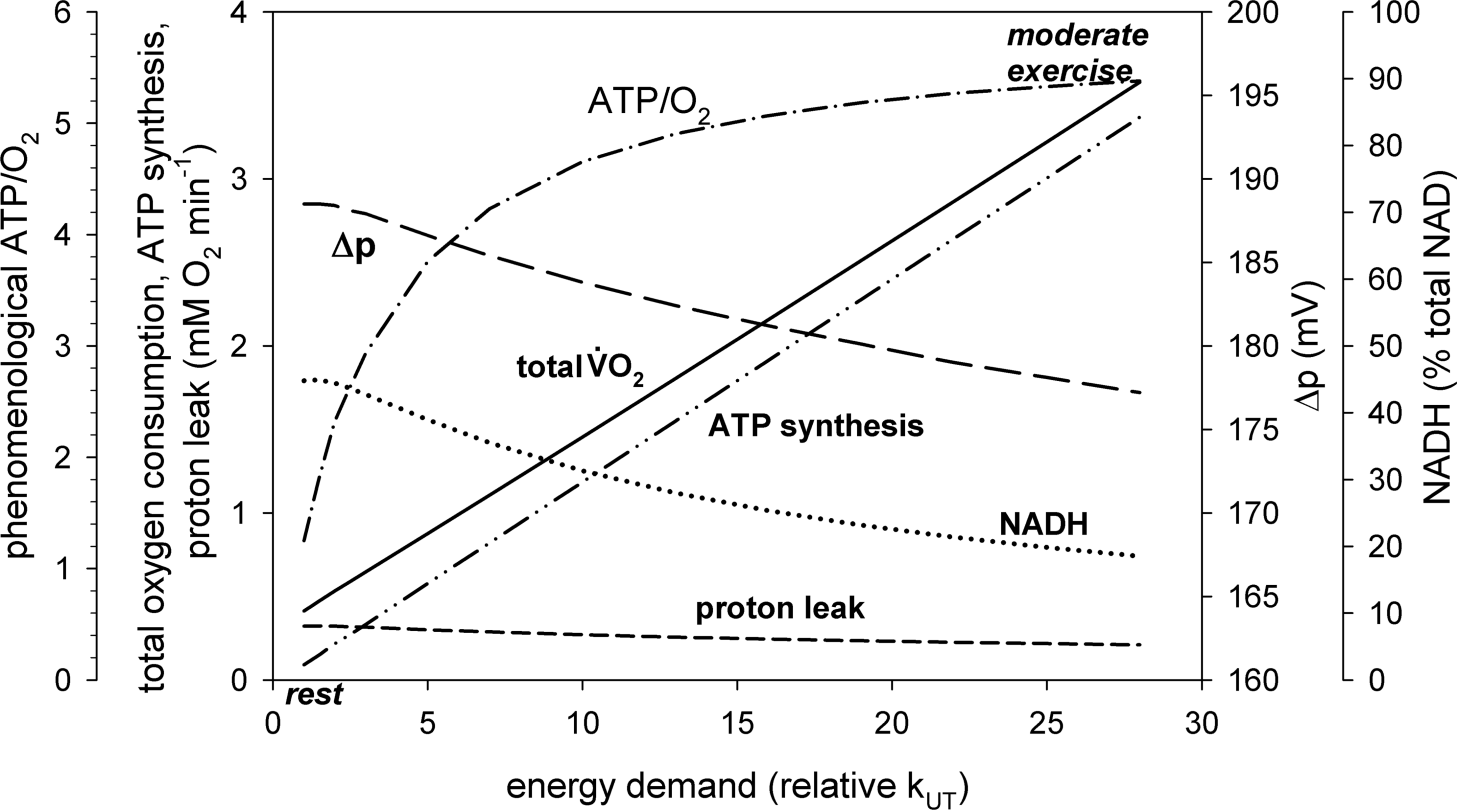
Dependence of total oxygen consumption ($\dot{V}O_{2}$), $\dot{V}O_{2}$ related to proton leak, $\dot{V}O_{2}$ related to ATP synthesis, Δp, NADH (% of total NAD) and phenomenological ATP/O_2_ ratio on relative activity of ATP usage (relative rate constant, k_UT_, or its activation in relation to rest, A_UT_) in skeletal muscle for proton leak activity (rate constant) elevated twice in relation to the ‘standard’ value.

**Fig 5:**
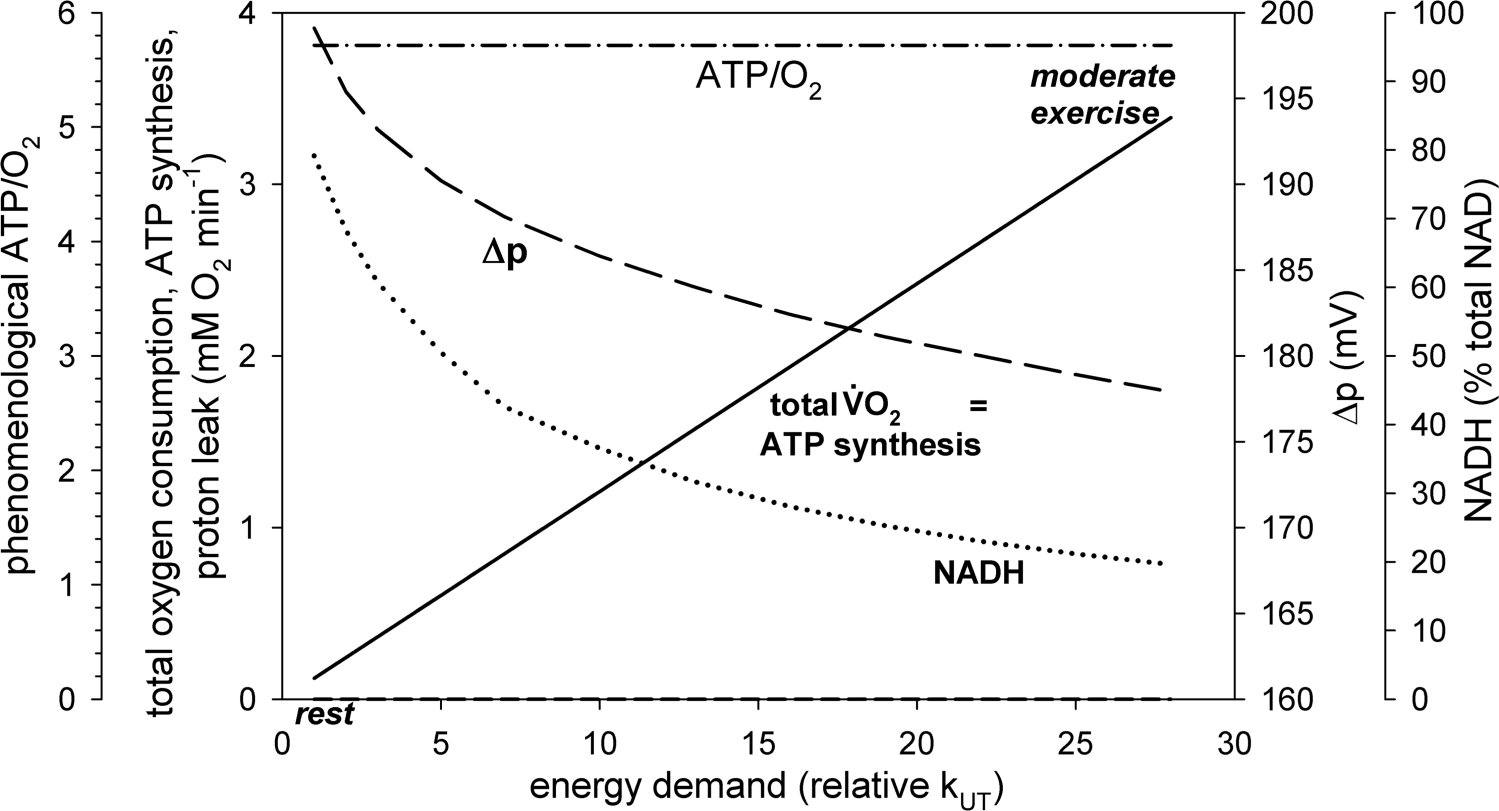
Dependence of total oxygen consumption ($\dot{V}O_{2}$), $\dot{V}O_{2}$ related to proton leak, $\dot{V}O_{2}$ related to ATP synthesis, Δp and NADH (% of total NAD) and phenomenological ATP/O_2_ ratio on relative activity of ATP usage (relative rate constant, k_UT_, or its activation in relation to rest, A_UT_) in skeletal muscle for proton leak activity (rate constant) set to zero (no proton leak).

The simulations for different work intensities (Figs 2–5) were made by a gradual increase in subsequent simulations of the rate constant of ATP usage (hydrolysis) k_UT_. The relative increase in k_UT_ (A_UT_, activation of ATP usage in relation to rest) varied from A_UT_ = 1 times at rest ($\dot{V}O_{2}$ = 0.27 mM min^-1^) to A_UT_ = 28 times for moderate work (resulting in $\dot{V}O_{2}$ of about 3.5 mM min^-1^) and A_UT_ = 80 times for heavy/severe work (resulting in $\dot{V}O_{2}$ of about 8.7 mM min^-1^). Therefore, A_UT_ was the measure or determinant of the work intensity, proportional to ATP usage for mechanical work. At the same time the activities (rate constants) of all OXPHOS complexes and NADH supply were elevated A_OX_ = A_UT_^0.35^ times. For instance, A_OX_ = 3.2 for moderate exercise (A_UT_ = 28) and A_OX_ = 4.6 for heavy/severe exercise (A_UT_ = 80). This corresponds to the each-step activation (ESA) mechanism of the regulation of OXPHOS during work transitions postulated previously [15–21] (the power coefficient p is the measure of ESA intensity; p = 0.35 means moderate ESA). Or, more precisely, this corresponds to the so-called mixed mechanism, where all OXPHOS complexes are directly activated (ESA), but to a smaller extent than ATP usage, and therefore the regulation by the negative feedback through elevated ADP and P_i_ co-operates with the regulation by ESA [19,24]. Glycolysis was activated (its rate constant was elevated) A_GL_ = A_UT_^0.7^ times [23]. In the ‘standard’ model version (Figs 2 and 3) the contribution of proton leak to $\dot{V}O_{2}$ at rest equaled 63%. It was assumed that ESA concerns OXPHOS complexes, but not proton leak. ESA is supposed to be a special mechanism that elevates the ATP production rate, and a potential activation of proton leak by ESA would be counter-productive (proton leak decreases Δp and thus ATP synthesis rate). Proton leak is not stimulated in isolated mitochondria by Ca^2+^ that can activate about twice all OXPHOS complexes (the Ca^2+^-induced elevated state 4 respiration was due to elevated protonmotive force, and not to direct activation of proton leak) [25].

The parameter values in the set of simulations presented in Figs 2 and 3 represent the ‘standard’ or ‘reference’ conditions. In the set of simulations shown in Fig 4 the rate constant (activity) of proton leak k_LK1_ was doubled in relation to the ‘standard’ set of simulations. This resulted in an increase in the contribution of proton leak to $\dot{V}O_{2}$ at rest to 78%. In the set of simulations shown in Fig 5 the rate constant of proton leak k_LK1_ was set to zero (no proton leak). Therefore, the contribution of proton leak to $\dot{V}O_{2}$ at rest was of course 0%.

At heavy/severe exercise in skeletal muscle the ‘additional’ ATP usage that is a major factor underlying the slow component of the $\dot{V}O_{2}$ on-kinetics [20] was omitted in computer simulations in order to achieve a steady-state.

Generally, within the model the proton leak-related $\dot{V}O_{2}$ (or vLK expressed in $\dot{V}O_{2}$ units) and ATP synthesis-related $\dot{V}O_{2}$ (or vAS expressed in $\dot{V}O_{2}$ units) are related to vLK and vAS in such a way that 4 electrons (e^-^) are used for the reduction of 1 O_2_ molecule, 20 H^+^ ions are pumped for 4 e^-^ flowing through the respiratory chain and 3.5 cytosolic H^+^ ions are used for synthesis of 1 cytosolic ATP molecule (2.5 H^+^ ions for synthesis of 1 matrix ATP molecule and 1 H^+^ ion for transport of ATP to cytosol and of ADP and P_i_ to mitochondrial matrix). Thus, vLK-related $\dot{V}O_{2}$ equals vLK / 20 and vAS-related $\dot{V}O_{2}$ equals vAS * 3.5 / 20.

## Theoretical results

### Proton leak-Δp dependence

The dependence of the proton leak intensity (vLK) on Δp in intact skeletal muscle used in the model (see Eq 1) is presented in Fig 1. In accordance with experimental data this dependence is strongly non-linear (non-ohmic). Particular states in skeletal muscle mitochondria are indicated in the diagram: state 4 (Δp = 195.4 mV), state 3 (Δp = 154.1 mV), rest state (Δp = 191.9 mV), moderate work state (Δp = 177.5 mV) and heavy/severe work state (Δp = 169.0 mV). The detailed relation between these states was discussed in a recent article [18]. It should be stressed that state 4 and state 3 in intact skeletal muscle is not the same as state 4 and state 3 in isolated mitochondria. First, vLK can be larger is isolated mitochondria than in skeletal muscle (for the same mitochondria volume/amount), as the inner mitochondrial membrane can be damaged in some fraction of mitochondria during mitochondria preparation. Second, usually a high constant P_i_ concentration is used in the isolated mitochondria system, while P_i_ level changes between different states in intact skeletal muscle [18].

### Δp, NADH, vLK-related $\dot{V}O_{2}$, contribution of vLK-related $\dot{V}O_{2}$ to total $\dot{V}O_{2}$ and ATP/O_2_ at rest, moderate work and heavy/severe work in intact skeletal muscle

Computer simulations predict that when work intensity increases in skeletal muscle Δp, NADH, proton leak intensity (vLK-related $\dot{V}O_{2}$) and its contribution to $\dot{V}O_{2}$ decrease, while the phenomenological ATP/O_2_ ratio increases. This decrease/increase is greater, the larger the work intensity. This can be seen in Figs 2 and 3. At rest, proton leak accounts for 63% of $\dot{V}O_{2}$. This is in agreement with the proton leak contribution to $\dot{V}O_{2}$ estimated for 60% in rat skeletal muscle [8] (see Table 1 therein). The contribution of proton leak to $\dot{V}O_{2}$ decreases dramatically with an increase in work intensity, as $\dot{V}O_{2}$ related to ATP production increases significantly due to the huge increase in the ATP usage activity. OXPHOS complexes are stimulated by ESA as well as by an increase in ADP and P_i_. At the same time the absolute proton leak intensity decreases due to the decrease in Δp (compare Eq 1). At moderate work (ATP usage activation A_UT_ = 28 times) proton leak accounts for only about 3% of $\dot{V}O_{2}$, while at heavy/severe work (A_UT_ = 80 times) it accounts for only about 1% of $\dot{V}O_{2}$. The presence of proton leak causes that the phenomenological ATP synthesis (vAS)-ADP relationship is significantly steeper (of higher phenomenological order) than the phenomenological $\dot{V}O_{2}$-ADP relationship, as can be seen in Fig 2.

Within the model the mechanistic (not involving proton leak) ATP/O_2_ ratio equals 5.71 (= 20/3.5: 20 protons pumped per 4 electrons or 1 O_2_, 3.5 protons needed for ATP synthesis and transport, see above). The phenomenological (involving proton leak) ATP/O_2_ ratio increases from about 2.1 at rest (A_UT_ = 1) to about 5.5 during moderate exercise (A_UT_ = 28) and about 5.7 during heavy/severe exercise (A_UT_ = 80). This is demonstrated in Fig 3.

For the parameter values used in the simulations shown in Fig 3 NADH decreases with an increase in work intensity. However, NADH can either decrease or increase during rest-to-work transition depending on how strongly NADH supply is directly activated or, more precisely, what is the balance of activation of the NADH-producing block and NADH-consuming block (OXPHOS + ATP usage). NADH can increase when the NADH-producing block is stimulated to a greater extent than the NADH-consuming block [21].

The estimation of the contribution of proton leak to $\dot{V}O_{2}$ equals to about 60% at rest in rat skeletal muscle [8]. However, it is most probably different in different animals, being greater in smaller animals. It can be potentially affected by some factors, such as thyroid hormones, ROS or temperature. Finally, Marcinek and co-workers [14] measured essentially no proton leak in resting skeletal muscle. Therefore, in two subsequent simulations the effect of doubling and reducing to zero of the ‘standard’ proton leak activity (rate constant) was checked.

A two-fold increase in proton leak activity (rate constant) k_LK1_ causes a decrease in Δp and NADH, especially at rest, while the proton leak intensity (flux) vLK (vLK-related $\dot{V}O_{2}$) of course increases. The contribution of proton leak (vLK-related $\dot{V}O_{2}$) to total $\dot{V}O_{2}$ at rest increases to 78%. This can be seen in Fig 4. However, the effect on Δp and NADH is rather small, especially at work. The phenomenological ATP/O_2_ ratio decreases significantly to about 1.3 at rest, but only by about 3% and 1% in relation to the ‘standard’ proton leak activity during moderate and heavy/severe exercise, respectively.

A switching off of proton leak (decrease of k_LK1_ to zero) elevates Δp and NADH, especially at rest. This is demonstrated in Fig 5. Of course, in this case the contribution of proton leak to $\dot{V}O_{2}$ at rest and work is 0% and the phenomenological ATP/O_2_ ratio at all work intensities is identical and equal to the mechanistic ATP/O_2_ ratio (5.71). The phenomenological ATP/O_2_ ratio increases by only about 3% and 1% in relation to the ‘standard’ proton leak activity during moderate and heavy exercise, respectively.

## Discussion

In the present study a computer model of the muscle bioenergetic system was used to study the dependence of Δp, NADH, proton leak intensity (vLK) (vLK-related $\dot{V}O_{2}$), ATP synthesis intensity (vAS) (vAS-related $\dot{V}O_{2}$), contribution of vLK-related $\dot{V}O_{2}$ and vAS-related $\dot{V}O_{2}$ to total $\dot{V}O_{2}$ and phenomenological ATP/O_2_ ratio on work intensity in skeletal muscle.

The simulated contribution of vLK to $\dot{V}O_{2}$ during moderate and heavy/severe exercise in skeletal muscle was very small despite large contribution of vLK to $\dot{V}O_{2}$ at rest and during low exercise. This was mostly caused by the huge increase in the ATP usage intensity during rest-to-work transition and thus in $\dot{V}O_{2}$ related to ATP synthesis. OXPHOS complexes were stimulated by each-step activation (ESA) and by increase in ADP and P_i_. On the other hand, proton leak was not directly stimulated and its intensity decreased due to the drop in Δp.

### Δp and vLK in different states

First, it was shown, what could be intuitively expected and what has been already at least partly demonstrated in the experimental way [8], that the highest Δp and vLK were in state 4, the smallest–in state 3, while at rest and during exercise they adopted intermediate values (see Fig 2). Δp and vLK at rest were closest to state 4, while during heavy exercise–to state 3.

### $\dot{V}O_{2}$-ADP dependence vs. vAS-ADP dependence

The slopes (orders) of the phenomenological (involving ESA) $\dot{V}O_{2}$-ADP dependence and the phenomenological vAS-ADP dependence differed significantly, and this difference was due to proton leak. This is demonstrated in Fig 2. $\dot{V}O_{2}$ and vAS (expressed in oxygen consumption equivalents) diverged significantly at low ADP present at rest, where vLK (vLK-related $\dot{V}O_{2}$) was relatively very significant, while they started to converge at high ADP concentrations present during work, where vLK decreased.

### Proton leak contribution to $\dot{V}O_{2}$ and phenomenological ATP/O_2_ ratio in skeletal muscle

#### Proton leak contribution to $\dot{V}O_{2}$

The increase in the total $\dot{V}O_{2}$ and vAS-related $\dot{V}O_{2}$ as well as the decrease in vLK-related $\dot{V}O_{2}$ with an increase in the ATP usage activity (energy demand), proportional to mechanical work intensity, for ‘standard’ proton leak activity is shown in Fig 3. At rest proton leak was responsible for about 63% of $\dot{V}O_{2}$. On the other hand, during heavy/severe exercise the absolute value of vAS-related $\dot{V}O_{2}$ was tens-fold greater (due to ESA as well as ADP and P_i_ increase in relation to rest) than the proton leak-related $\dot{V}O_{2}$. At the same time, Δp decreased significantly with the increase in the ATP usage activity (energy demand). This caused a decrease in the absolute value of vLK (vLK-related $\dot{V}O_{2}$) at heavy work in relation to moderate work and the more in relation to rest.

#### Changes in Δp and NADH

Both Δp and NADH decreased with an increase in work intensity in the simulations shown in Figs 3–5. In skeletal muscle different elements of the ATP supply system are activated by ESA to a lower extent than ATP usage. As a result, ADP and P_i_ always increase, while Δp always decreases during rest-to-work transition. Δp is related to the mitochondrial ATP/(ADP*P_i_) ratio (through ATP synthase) and cytosolic ATP/(ADP*P_i_) ratio (through ATP/ADP carrier and P_i_ carrier). Therefore, the decrease in (cytosolic and mitochondrial) ATP/(ADP*P_i_) resulting from the increase in ADP and P_i_ implies that Δp also decreases.

On the other hand, NADH can either increase or decrease during rest-to-work transition in skeletal muscle, depending on the balance of the relative direct activation of the NADH-supply block and NADH-consuming block (OXPHOS + ATP usage) [21]. When the NADH-producing block is activated to a greater extent than the NADH-consuming block, NADH increases during rest-to-work transition [21].

#### Proton leak contribution to $\dot{V}O_{2}$—standard conditions

Of course, the huge increase in vAS-related $\dot{V}O_{2}$ and decrease in the vLK-related $\dot{V}O_{2}$ with the work intensity increase resulted in a decrease in the contribution of vLK-related $\dot{V}O_{2}$ to total $\dot{V}O_{2}$. While it was as high as about 60% at rest (in a good agreement with experimental data [8]), it dropped to about 3% during moderate exercise and to about 1% during heavy/severe exercise. This was associated with an increase in the phenomenological (involving proton leak) ATP/O_2_ ratio (the mechanistic, not involving proton leak, ATP/O_2_ ratio equals 5.71 within the model). It rised from about 2.1 at rest to about 5.5 during moderate exercise and about 5.7 during heavy/ severe exercise (see Fig 3). Therefore, the muscle coupling efficiency (the ratio of the phenomenological ATP/O_2_ to the mechanistic ATP/O_2_) increased with the work increase (and approached 1), given that all other factors remained unchanged. An increase in the ATP/O_2_ ratio with an increase in $\dot{V}O_{2}$ was observed in skeletal muscle mitochondria [26], where OXPHOS is activated by an increase in ADP between state 4 and state 3, while vLK decreased due to a decrease in Δp. A similar effect was observed in permeabilized skeletal muscle myofibers: the ATP/O ratio (which is a half of the ATP/O_2_ ratio) was less than one for low ADP concentration (15 μM) and thus low $\dot{V}O_{2}$, but exceeded two for moderate (200 μM) and maximal (2000 μM) ADP concentration and $\dot{V}O_{2}$ [27].

The very low contribution of proton leak to $\dot{V}O_{2}$ and, consequently, high phenomenological ATP/O_2_ ratio in intensively working skeletal muscle was mostly caused by the huge increase in the ATP usage activity between rest and moderate and heavy/severe exercise and by the fact that ESA concerned particular OXPHOS complexes, but not proton leak. ESA increased very significantly the absolute value of the vAS-related $\dot{V}O_{2}$, while it did not affect the vLK-related $\dot{V}O_{2}$. Additionally, the vAS-related $\dot{V}O_{2}$ was stimulated by elevated ADP and P_i_. On the other hand, the absolute vLK-related $\dot{V}O_{2}$ decreased in working skeletal muscle in relation to rest due to the decrease in Δp. Therefore, the phenomenological (involving proton leak) ATP/O_2_ ratio during intensive muscle work was very close to the mechanistic (not involving proton leak) ATP/O_2_ ratio, and the muscle coupling efficiency (defined as the ratio of the phenomenological ATP/O_2_ ratio to the mechanistic ATP/O_2_ ratio) remained very high (very close to 1).

#### Effect of proton leak activity (rate constant)

Elevated thyroid hormones level [10], muscle training (increase in UCPs sensitivity to fatty acids [28]), increased temperature [29] and/or increased ROS concentration (activating UCPs [13]) can all elevate the proton leak intensity. Therefore, the effect of doubling of proton leak activity (rate constant) was simulated. The theoretical results are presented in Fig 4. One can see that the increase in the proton leak activity (rate constant k_LK1_) elevated vLK-related $\dot{V}O_{2}$ and the total $\dot{V}O_{2}$. As a result, the contribution of proton leak to $\dot{V}O_{2}$ at rest rised to 78%. On the other hand, the 2-fold activation of proton leak diminished Δp and NADH, especially at rest. This was caused by accelerated Δp dissipation. It also decreased muscle coupling efficiency (related to the phenomenological ATP/O_2_ ratio) during exercise. However, in the ‘standard’ simulation without proton leak activation (Fig 3) the contribution of vLK-related $\dot{V}O_{2}$ to total $\dot{V}O_{2}$ was very low: about 3% and 1% during moderate and heavy/severe exercise, respectively. A two-fold activation of proton leak elevated these values to 6% and 2%, respectively. As a result, even doubling of vLK decreased the phenomenological ATP/O_2_ ratio only by about 3% and 1%, respectively.

When proton leak was ‘switched off’ (its rate constant k_LK1_ was set to zero), the total $\dot{V}O_{2}$ decreased, as it was equal then to vAS-related $\dot{V}O_{2}$. This was related to an increase in Δp and NADH, especially at rest, as can be seen in Fig 5. In this case, the phenomenological (involving proton leak) ATP/O_2_ ratio increased (in relation to the case with ‘standard’ proton leak activity) only by about 3% and 1% during moderate and heavy/severe exercise, respectively. The phenomenological ATP/O_2_ ratio was the same at all work intensities and equaled the mechanistic ATP/O_2_ ratio.

Generally, large changes in the proton leak activity (rate constant k_LK1_) seemed to have only a minor impact on the phenomenological ATP/O_2_ ratio and muscle coupling efficiency during intense exercise. This was valid for such a broad range of the proton leak contribution to $\dot{V}O_{2}$ at rest as about 0–80%. Therefore, the exact determination of this contribution [8] is not very important in this context, although it is of cause very important in the context of thermogenesis at rest and basal metabolic rate.

### vLK-related $\dot{V}O_{2}$ contribution to total $\dot{V}O_{2}$ and ATP/O_2_ in isolated mitochondria vs. intact working muscle

Isolated mitochondria, at least in the absence of Ca^2+^, lack ESA. Therefore, $\dot{V}O_{2}$ cannot be elevated here as much as in intact skeletal muscle. Additionally, the inner mitochondrial membrane can be damaged in some fraction of mitochondria during the isolation procedure. This would elevate state 4 respiration related to proton leak. For these reasons it can be expected that the contribution of vLK-related $\dot{V}O_{2}$ to total $\dot{V}O_{2}$ is much smaller in intensively working muscle than in isolated mitochondria and the ATP/O_2_ ratio is somewhat higher. For this reason, the experimental measurements of the relative proton leak intensity (e.g., of the respiratory control ratio, RCR) and ATP/O_2_ in isolated mitochondria, although very valuable for many purposes, cannot be directly extrapolated to intact working muscle.

### Phenomenological ATP/O_2_ ratio and ATP supply by anaerobic glycolysis

The term ‘phenomenological ATP/O_2_ ratio’ used throughout the present article means ‘involving proton leak’, as opposed to ‘mechanistic ATP/O_2_ ratio’, not involving proton leak. However, it should be stressed that the term ‘phenomenological ATP/O_2_ ratio’ means in fact ‘oxidative phenomenological ATP/O_2_ ratio’. As, in intact muscle, especially during heavy/severe exercise, where ATP supply by both aerobic and anaerobic glycolysis is present, the ‘overall’ or ‘observed’ ‘phenomenological ATP/O_2_ ratio’ is higher than the ‘oxidative phenomenological ATP/O_2_ ratio’, related only to ATP production by OXPHOS. The overall phenomenological ATP/O_2_ ratio can be even higher than the mechanistic ATP/O_2_ ratio for OXPHOS. This fact should be always kept in mind when speaking about the ATP/O_2_ ratio and it should be always specified which ATP/O_2_ ratio is meant in a given case. On the other hand, any non-mitochondrial oxygen consumption (for instance by the antioxidant system) (see ref. 8) decreases the overall phenomenological ATP/O_2_ ratio. It is postulated here to distinguish three sorts of the ATP/O_2_ ratio: 1. mechanistic ATP/O_2_ ratio for OXPHOS without proton leak; 2. oxidative phenomenological ATP/O_2_ ratio for oxidative ATP supply involving of proton leak; 3. overall phenomenological ATP/O_2_ ratio for oxidative (in the presence of proton leak) and glycolytic (by aerobic and anaerobic glycolysis) ATP supply, taking into account the non-mitochondrial oxygen consumption.

### Study limitations

It must be stressed that even a well-tested computer model can be only an approximation of the complex reality.

It is likely that the relative contribution of vLK-related $\dot{V}O_{2}$ to total $\dot{V}O_{2}$ at rest in bigger mammals (including humans) is lower than in rats. This is because the proton leak intensity is inversely proportional to the mammal body mass [11] and bigger mammals need less thermogenesis per body mass in order to maintain high constant body temperature. Proton leak intensity can be affected by exercise-induced muscle temperature and/or cytosolic ROS increase. Therefore, the strictly quantitative theoretical predictions obtained in the present study should be treated with some caution. Nevertheless, these limitations do not affect the general conclusion drawn in the present study.

## Conclusions

Computer simulations demonstrated that while the contribution of the proton leak through the inner mitochondrial membrane to VO_2_ was large at rest and at low work, it decreased progressively with work intensity and became very small at moderate and heavy/severe work. The absolute value of the proton leak flux (vLK) and vLK-related VO_2_ decreased together with an increase in work intensity in skeletal muscle, which was caused by a decrease in Δp, given that such factors as ROS or elevated temperature do not stimulate it significantly. On the other hand, the ATP synthesis rate (vAS) and vAS-related VO_2_ increased linearly with work intensity (ATP demand activity). While in resting skeletal muscle at ‘standard’ proton leak activity the contribution of vLK-related $\dot{V}O_{2}$ to total $\dot{V}O_{2}$ amounted about 60%, this contribution dropped to about 3% during moderate exercise and 1% during heavy/severe exercise. This was due to a huge increase in the ATP usage activity and thus in the ATP synthesis-related $\dot{V}O_{2}$. The great increase in $\dot{V}O_{2}$ during rest-to-work transition was directly related to each-step activation, ESA, of OXPHOS complexes and to activation of OXPHOS by ADP and P_i_ increase. Proton leak was not activated directly and its absolute value decreased due to the decrease in Δp. As a result, the contribution of LK-related VO_2_ to total $\dot{V}O_{2}$ decreased very significantly during rest-to-intensive work transition. This was associated with a significant increase in the phenomenological (involving proton leak) ATP/O_2_ ratio. A two-fold increase in the activity (rate constant) of proton leak increased the contribution of vLK-related $\dot{V}O_{2}$ to total $\dot{V}O_{2}$ to about 80% at rest, about 6% at moderate exercise and about 2% at heavy/severe exercise. A removal of proton leak (reduction of its activity to zero) was associated with an increase in the phenomenological ATP/O_2_ ratio by about 3% during moderate exercise and 1% during heavy/severe exercise in relation to ‘standard’ proton leak activity. Therefore, even large variations in the proton leak activity have only a small impact on the system properties during moderate and heavy/severe exercise (although they have a big effect at rest and during low exercise). In other words, whatever the (realistic) contribution of proton leak to $\dot{V}O_{2}$ at rest, it is small during intense work.
